# Dynamical behavior of Borospherene: A Nanobubble

**DOI:** 10.1038/srep11287

**Published:** 2015-06-22

**Authors:** Gerardo Martínez-Guajardo, José Luis Cabellos, Andres Díaz-Celaya, Sudip Pan, Rafael Islas, Pratim K. Chattaraj, Thomas Heine, Gabriel Merino

**Affiliations:** 1 Departamento de Física Aplicada, Centro de Investigación y de Estudios Avanzados, Unidad Mérida. Km 6 Antigua Carretera a Progreso. Apdo. Postal 73, Cordemex, 97310, Mérida, Yuc., México; 2 Unidad Académica de Ciencias Químicas, Área de Ciencias de la Salud, Universidad Autónoma de Zacatecas, Km. 6 carretera Zacatecas-Guadalajara s/n, Ejido La Escondida C. P. 98160, Zacatecas, Zac., México; 3 Department of Chemistry and Center for Theoretical Studies, Indian Institute of Technology Kharagpur, 721302, India; 4 Departamento de Ciencias Químicas, Facultad de Ciencias Exactas Universidad Andres Bello, República 275, Santiago, Chile; 5 Center of Functional Nanomaterials (NanoFun), School of Engineering and Science, Jacobs University, Bremen, Bremen, 28759, Germany

## Abstract

The global minimum structure of borospherene (B_40_) is a cage, comprising two hexagonal and four heptagonal rings. Born-Oppenheimer Molecular Dynamics simulations show that continuous conversions in between six and seven membered rings take place. The activation energy barrier for such a transformation is found to be 14.3 kcal·mol^−1^. The completely delocalized σ- and π-frameworks, as well as the conservation of the bonding pattern during rearrangement, facilitate the dynamical behavior of B_40_. B_40_ is predicted to act as a support-free spherical two-dimensional liquid at moderate temperature. In other words, B_40_ could be called as a nanobubble.

Is it possible to build a buckyball comprising only boron atoms? Boron is an electron deficient atom with only three valence electrons. So, a perfect boron buckminsterfullerene (B_60_) is not expected to be stable owing to the absence of the fourth electron, which is essential for the π-stabilization of a spherical shell. A fullerene-like boron cluster, B_80_, was predicted *in silico*, which has structural similarity with C_60_ but with an additional boron atom at the center of each hexagon^1^. However, several unsymmetrical B_80_ structures were found to be more stable than the fullerene-like geometry^2^,^3^,^4^,^5^,^6^.

Quite recently, Zhai *et al.*^7^ reported the experimental detection of B_40_^−^ by photoelectron spectroscopy. Density functional theory (DFT) computations revealed that the most stable structure includes a quasi-planar arrangement with two adjacent hexagonal holes. However, a cage structure is also viable as the relative energy to the global minimum is only 1.7 kcal·mol^−1^. In contrast, the lowest energy structure of the neutral B_40_ cluster possesses a perfect cage-like shape containing two hexagonal and four heptagonal holes (**1**, see Fig. 1) and it is more stable than the corresponding quasi-planar form by approximately 27 kcal·mol^−1^. The bonding network in B_40_ is completely delocalized via σ– and π-type multicenter bonds. The structure gains its stability due to its very high HOMO-LUMO energy gap (3.13 eV), which is comparable to that of C_60_ (3.02 eV). So, B_40_ represents the first pure boron buckyball (borospherene). Very recently, a couple of boron buckyballs (B_38_ and B_39_^−^) were also reported in the literature^8^,^9^.

Four years ago, the B_19_^−^ cluster was reported to have a perfect planar structure with a central filled pentagonal unit inside a B_13_ ring. Some of us subsequently found a remarkable fluxional behavior in the B_19_^−^ cluster^10^,^11^. B_19_^−^ exhibits an almost free rotation of the internal pentagon-shaped hub within the co-planar B_13_ ring akin to a Wankel motor, which is evident from the Born-Oppenheimer Molecular Dynamics (BO-MD) simulations. In fact, the zero-point energy is sufficient to overcome the barrier that allows the inner and outer rings to rotate independent of each other. The presence of various multicenter bonds between the outer-ring and the inner-ring, that could easily migrate from one position to another during rotation, facilitates such a dynamical feature^12^. This fluxional behavior is not limited to B_19_^−^ only, B_13_^+^,^13^,^14^,^15^ B_18_^2−^,^16^,^17^ and B_20_^−^ also show similar dynamical features^18^,^19^. As B_40_ has a similar multicenter bonding pattern, there arises the compelling question, “Is B_40_ also fluxional?” and that prompted us to explore the probable dynamical behavior of B_40_. Indeed, a fluxional behavior of a cage would correspond to a nanobubble — a molten, hollow pure boron object, which is unprecedented to our knowledge, and thus is of immense interest concerning its chemical and physical properties.

In order to explore the dynamical behavior of B_40_, we carried out a series of BO-MD simulations at the PBE/DZVP^20^ level in deMon2K (deMon2k v. 3, the deMon developers, Cinvestav, Mexico City 2011). The simulations are launched from the equilibrium geometry of B_40_ (**1**) with random velocities assigned to the atoms, employing a Hoover thermal bath, for a simulation time of 25 ps with 0.5 fs time steps. During the MD computations, we keep the total angular momentum of the cluster as zero, thereby suppressing the cluster rotation. The behavior of the mean square displacement (msd) as a function of time easily allows us to differentiate between a solid-like and a liquid-like behavior. The mean-square displacement at time t is given by


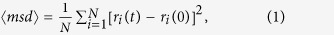


where *r*_i_(*t*) is the position vector of the i-*th* atom at the time t and N is the total number of atoms in the system.

During the BO-MD simulations at 1000 K, the cluster maintains its connectivity pattern and cage-like structure as was reported by Zhai *et al.*^7^ But at 1200 K and 1500 K, a continuous transformation between the seven membered rings (7-MRs) and six membered rings (6-MRs) is perceived (see movie files in the electronic supporting information). The structure of the rear 7-MR first gets distorted by moving one B atom towards the adjacent 6-MR and then it becomes a 6-MR, which transforms the contiguous 6-MR into a 7-MR. But still the near 7-MR does not show any significant structural change. Thereafter, the rear 6-MR is again transformed into 7-MR and the near 7-MR starts to have a structural deformation. Therefore, the boron cage certainly shows a series of transformation in which the ring sizes continuously get changed during the simulations.

Figure 1 depicts the minimum energy structures and the transition state involved in the structural change of B_40_ (see the Cartesian coordinates in the Supplementary Information), which were optimized at the PBE0/6-311+G(d)^21^ level using Gaussian 09 program (Gaussian 09 Revision D.1, Wallingford CT, 2009). The global minimum corresponds to a *D*_2*d*_ structure, such that the 6-MRs are in front of each other. The transition state (**TS1**) related to the transformation between a 7-MR and a 6-MR has a *C*_*s*_ geometry (υ_min_ = 117*i* cm^−1^). It is only 14.3 kcal·mol^−1^ higher in energy (including the zero-point energy correction) than **1**. The change comprises a B_5_ fragment located in between a 6-MR and a 7-MR, which contains a quasi-planar tetracoordinate boron atom. The result of this alteration is the intermediate cage **2** (with *C*_*s*_ symmetry), which possesses two adjacent 6-MRs. The relative energy between **1** and **2** is only 11.1 kcal·mol^−1^.

Structure **2** can return to **1** via the same transition state. In Fig. 1, two boron atoms are labeled as B1 and B2 in order to follow the transformations. These atoms form part of the B_5_ fragment, which suffers the relevant changes. Originally B1 and B2 belong to a 7-MR. After the transformation into **2**, B1 becomes a part of a 6-MR and B2 is moving to form a new 7-MR. Note that there are four B_5_ fragments surrounding the two 6-MRs and all of them are suitable for such a structural reorganization. If the central B_5_ moiety changes, the original structure is recovered. In contrast, if any of the other B_5_ fragments switches, then four boron atoms will change their positions to form a 7-MR from a 6-MR.

Real-temperature simulations on realistic time scales are computationally impossible for quantum-mechanical systems to date. As a typical time step is about one fs (10^−15^ s), 10^15^ MD steps would be required. Given that it is unrealistic from the computational point of view, the standard procedure to overcome this problem is to run simulations at higher temperature, what essentially serves as a time lapse. In our case, the rare event is overcoming a barrier of 14.3 kcal/mol. Using Boltzmann’s law, our 25 ps simulation at 1200 K corresponds roughly to 0.5 ms in reality. Thus, with in this – admittedly crude – approximation we do observe fluxionality at chemically relevant time scales. In other words, it does not mean that the transformation occurs at 1200 K, it is only that at this temperature, the molecule has the enough kinetic energy to cross the barrier in a 25 ps time frame.

The extraordinary dynamical behavior of B_40_ is also reflected in the 〈msd〉 values (Fig. 2). In a solid-like system, one would expect the 〈msd〉 to be essentially a constant close to zero, whereas a liquid-like system should exhibit a nearly linear increase in 〈msd〉 with respect to time^22^. At 1000 K, clearly B_40_ behaves like a solid-like system, where the structure remains almost the same during the MD simulation, but the behavior of <msd> changes drastically at 1200 K and 1500 K. As a result of the nuclear mobility, B_40_ can be described as a surface or two-dimensional liquid-like system at these temperatures. Thus, B_40_ is a system that behaves like a support-free spherical two-dimensional liquid.

Fluxionality in carboranes is common, but no such interchange in the sizes of the rings occurs there. On the other hand, the classical carbon cages like C_60_ do not show such type of transformations. A somewhat similar process is the formation of Stone-Wales (SW) defects^23^ in fullerenes or nanotubes. However, particularly for C_60_, this isomerization is restricted due to a very high activation energy barrier (approximately 7 eV in fullerenes). So, the barrier in B_40_ is significantly lower than that in the SW defects formation in carbon systems. In other words, B_40_ is the first boron cluster that shows an interesting dynamical behavior with a moderate barrier (less than 15 kcal/mol), which allows that the ring sizes get changed continuously.

The bonding situation in B_40_ is intriguing. Zhai *et al.*^7^ studied in detail the nature of bonding in **1** using the adaptive natural density partitioning (AdNDP) analysis^24^ and showed that a strong delocalization of the σ- and π-system is present in **1**. 48 pairs of valence electrons are delocalized via σ-bonds and the remaining electrons are delocalized via π-bonds. Zhai *et al.*^7^ also computed the nucleus independent chemical shift (NICS)^25^ to determine whether B_40_ could be classified as an aromatic system. NICS indicates that the system is indeed aromatic in nature. However, there are some details about delocalization that were not explored previously and could provide some insight into the fluxional behavior of **1**. Here we use the induced magnetic field, particularly the z-component of the induced magnetic field (*B*^ind^_z_)^26^,^27^, in order to understand delocalization.

Figure 3 shows the profiles of *B*^ind^_z_ for external fields applied perpendicular to the 6-MRs. An external magnetic field in this direction can induce a current around the cage. The magnetic response at the cage center is very high (*B*^ind^_z_ = −47.1 ppm) and diatropic in nature. This value is even larger than those computed in other spherenes^28^,^29^,^30^. The intensity of *B*^ind^_z_ in *B*_40_ diminishes gradually along the center to surface of the cage. The radius of B_40_ is approximately 2.5 Å. At this distance, the |*B*^ind^_z_| values are around 20 ppm lower than that computed at the cage center. However, at both 6-and 7-MRs, the magnitudes are still appreciable (*B*^ind^_z_ ≈ −20 ppm), indicating a strong delocalization in the σ-framework.

The *B*^ind^_z_ profile, which is mathematically equivalent to NICS_zz_, shows a long-range shielding cone above the 6-MRs with an extension of nearly 10 Å, which is even more intense than that computed at C_60_. In contrast, the magnetic response is different on the 7-MRs. In this case, the *B*^ind^_z_ values become positive at around 3.5 Å above, implying a decrease in delocalization above the 7-MRs. Hence, these results indicate a strong π-delocalization at the 6-MRs, which is much higher than those at the 7-MRs.

The *B*^ind^_z_ profiles of **TS1** and **2** show essentially the same magnetic responses in both, shape and intensity as that in **1** (see Fig. 1-SI). Thus, during the rearrangement process, the changes in the σ- and π-delocalizations are not significant. This is the main difference compared to the carbon cages like C_60_, where the changes in the connectivity (as Stone-Wales defects) modify the σ- and π-frameworks drastically and accordingly the delocalization and stability.

In summary, BO-MD simulations reveal that B_40_, the first pure all-boron buckyball, shows a fascinating dynamical behavior in which the ring sizes continuously get changed during simulation. The transformation between 6- and 7-MRs occurs through an activation energy barrier of 14.3 kcal·mol^−1^. The electronic structure of B_40_ favors such transformation as the σ- and π-delocalizations are properly maintained in both the minimum energy structures (**1** and **2**) as well as in the corresponding transition state (**TS1**). During the rearrangement, the changes around the multicenter B-B bonds are almost negligible, thus unlike in the carbon cages the barrier is not prohibitive. This is also related to the fact that a B-B bond is weaker than the C-C one. So, the dynamic behavior of B_40_ is a consequence of the strong delocalization existing around the multicenter B-B bonds. The msd supports that B_40_ is a nanobubble that behaves like a support-free spherical two-dimensional liquid.

## Additional Information

**How to cite this article**: Martínez-Guajardo, G. *et al.* Dynamical behavior of Borospherene: A Nanobubble. *Sci. Rep.* **5**, 11287; doi: 10.1038/srep11287 (2015).

## Supplementary Material

Supplementary Movie S1

Supplementary Movie S2

Supplementary Movie S3

Supplementary Information

## Figures and Tables

**Figure 1:**
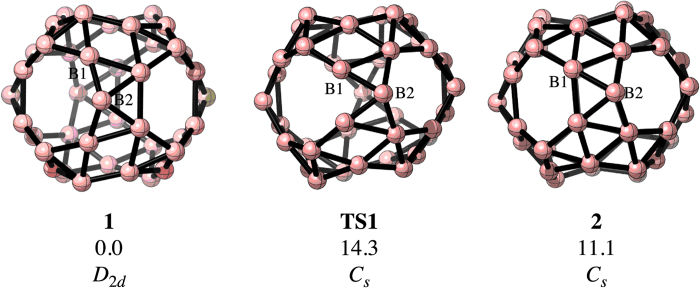
The minimum energy structures (1 and 2) and the transition state (TS1) involved in the fluxional behavior of B_40_. The relative energies (in kcal·mol^−1^) are computed at the PBE0/6-311+G(d) level, including the zero point energy correction.

**Figure 2:**
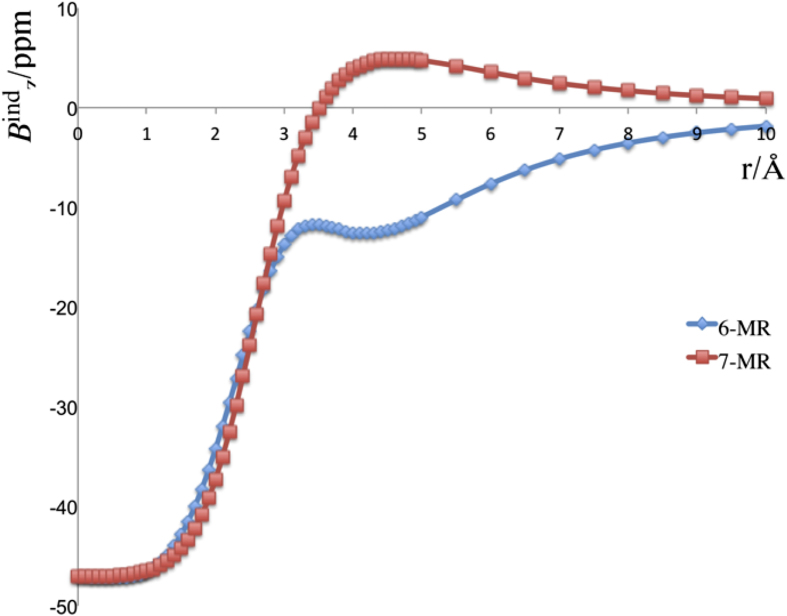
The profiles of the z-component of the induced magnetic field (B^ind^z) for 1. The blue (red) line shows the magnetic response computed when the external magnetic field is applied perpendicular to the 6-MR (7-MR). The scale is given in ppm (μT for |B^ext^| = 1 T).

**Figure 3:**
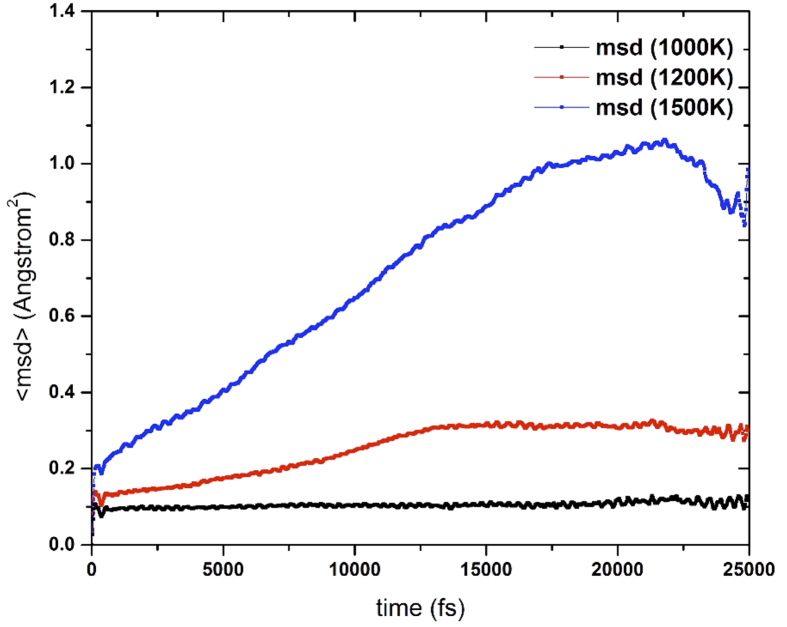
Temporal variation of the mean square displacements, , for B40 at 1000, 1200, and 1500 K.
