# Supplementary material for: Dynamical behavior of Borospherene: A Nanobubble
Source: Sci Rep. 2015 Jun 22;5:11287. doi: 10.1038/srep11287 (PMC4476142; doi:10.1038/srep11287)
Supplement: Supplementary Information [file srep11287-s4.pdf]

# Dynamical behavior of Borospherene: A Nanobubble

Gerardo Martínez-Guajardo,[1,2] José Luis Cabellos,[1] Andres Díaz-Celaya,[1] Sudip Pan,[3] Rafael Islas,[4] Pratim K. Chattaraj,[3] Thomas Heine,[5,*] and Gabriel Merino.[1,*]

[1] Departamento de Física Aplicada, Centro de Investigación y de Estudios Avanzados, Unidad Mérida. Km 6 Antigua Carretera a Progreso. Apdo. Postal 73, Cordemex, 97310, Mérida, Yuc., México.

[2] Unidad Académica de Ciencias Químicas, Área de Ciencias de la Salud, Universidad Autónoma de Zacatecas, Km. 6 carretera Zacatecas-Guadalajara s/n, Ejido La Escondida C. P. 98160, Zacatecas, Zac., México.

[3] Department of Chemistry and Center for Theoretical Studies, Indian Institute of Technology Kharagpur, 721302, India.

[4] Departamento de Ciencias Químicas, Facultad de Ciencias Exactas Universidad Andres Bello, República 275, Santiago, Chile.

[5] Center of Functional Nanomaterials (NanoFun), School of Engineering and Science, Jacobs University Bremen, Bremen, 28759, Germany.

gmerino@mda.cinvestav.mx

t.heine@jacobs-university.de

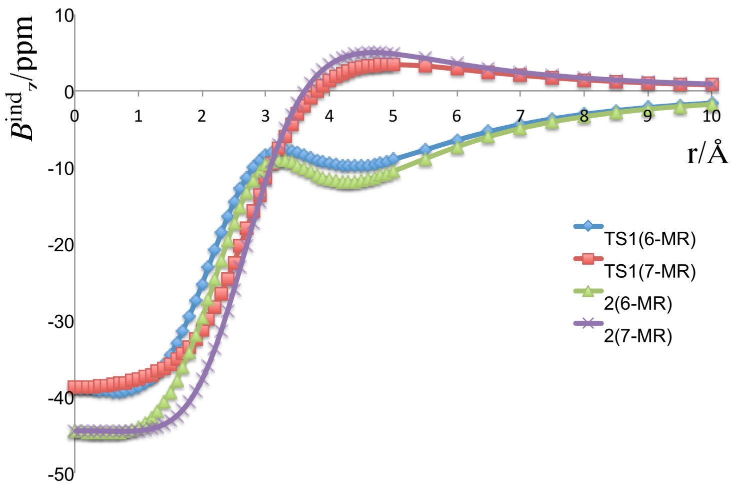


**Figure 1-SI**. The profiles of the z-component of the induced magnetic field ($B^{ind}_z$) for **TS1** and **2**. The profiles show the magnetic response computed when the external magnetic field is applied perpendicular to the 6-MR. The scale is given in ppm (μT for |$\mathbf{B}^{ext}$| = 1 T).

**Cartesian Coordinates**

**1**

| | | | |
|---|---|---|---|
| B | -3.161826000 | 0.695072000 | 0.382516000 |
| B | -1.191638000 | -1.191638000 | -2.862292000 |
| B | -2.652243000 | 0.689854000 | -1.202257000 |
| B | 3.161826000 | -0.695072000 | 0.382516000 |
| B | 1.191638000 | -1.191638000 | 2.862292000 |
| B | -1.865133000 | -1.865133000 | 1.983283000 |
| B | 2.293076000 | 1.055564000 | -1.678584000 |
| B | 0.689854000 | 2.652243000 | 1.202257000 |
| B | -0.695072000 | 3.161826000 | 0.382516000 |
| B | 1.191638000 | 1.191638000 | -2.862292000 |
| B | -0.689854000 | 2.652243000 | -1.202257000 |
| B | -0.695072000 | -3.161826000 | -0.382516000 |
| B | 0.689854000 | -2.652243000 | -1.202257000 |
| B | 2.652243000 | -0.689854000 | -1.202257000 |
| B | 2.293076000 | -1.055564000 | 1.678584000 |
| B | -2.293076000 | 1.055564000 | 1.678584000 |
| B | 1.055564000 | 2.293076000 | -1.678584000 |
| B | 1.055564000 | -2.293076000 | 1.678584000 |
| B | 1.865133000 | -1.865133000 | -1.983283000 |
| B | -3.161826000 | -0.695072000 | -0.382516000 |
| B | -2.293076000 | -1.055564000 | -1.678584000 |
| B | -1.055564000 | -2.293076000 | -1.678584000 |
| B | 2.652243000 | 0.689854000 | 1.202257000 |

| | | | |
|---|---|---|---|
| B | -2.652243000 | -0.689854000 | 1.202257000 |
| B | -0.689854000 | -2.652243000 | 1.202257000 |
| B | 1.609843000 | -0.355818000 | -2.536010000 |
| B | 0.355818000 | -1.609843000 | -2.536010000 |
| B | 0.695072000 | -3.161826000 | 0.382516000 |
| B | -0.355818000 | -1.609843000 | 2.536010000 |
| B | 1.865133000 | 1.865133000 | 1.983283000 |
| B | -1.055564000 | 2.293076000 | 1.678584000 |
| B | -0.355818000 | 1.609843000 | -2.536010000 |
| B | -1.609843000 | 0.355818000 | -2.536010000 |
| B | -1.609843000 | -0.355818000 | 2.536010000 |
| B | 3.161826000 | 0.695072000 | -0.382516000 |
| B | -1.865133000 | 1.865133000 | -1.983283000 |
| B | 1.609843000 | 0.355818000 | 2.536010000 |
| B | -1.191638000 | 1.191638000 | 2.862292000 |
| B | 0.695072000 | 3.161826000 | -0.382516000 |
| B | 0.355818000 | 1.609843000 | 2.536010000 |

**2**

| | | | |
|---|---|---|---|
| B | 1.828199000 | 0.495283000 | 2.407466000 |
| B | 1.522758000 | -3.008799000 | 0.000000000 |
| B | 1.391376000 | -1.185096000 | 2.251929000 |
| B | -1.885847000 | 0.469119000 | -2.787763000 |
| B | 0.164791000 | 2.749625000 | -1.757607000 |
| B | 2.478897000 | 2.209829000 | 0.000000000 |
| B | -2.511004000 | -1.545968000 | -0.876763000 |
| B | -2.310446000 | 1.348903000 | 1.430292000 |
| B | -1.885847000 | 0.469119000 | 2.787763000 |
| B | -1.836183000 | -2.722041000 | 0.000000000 |
| B | -1.480210000 | -1.079237000 | 2.350527000 |
| B | 2.807778000 | -0.563698000 | -1.685397000 |
| B | 1.391376000 | -1.185096000 | -2.251929000 |
| B | -1.480210000 | -1.079237000 | -2.350527000 |
| B | -0.901858000 | 1.688930000 | -2.456823000 |
| B | 0.748364000 | 1.630090000 | 2.719965000 |
| B | -2.511004000 | -1.545968000 | 0.876763000 |
| B | 0.748364000 | 1.630090000 | -2.719965000 |
| B | -0.063978000 | -1.811099000 | -2.575742000 |
| B | 2.807778000 | -0.563698000 | 1.685397000 |
| B | 2.289736000 | -1.873019000 | 0.876778000 |
| B | 2.289736000 | -1.873019000 | -0.876778000 |
| B | -2.310446000 | 1.348903000 | -1.430292000 |
| B | 2.721591000 | 0.862899000 | 0.884755000 |
| B | 2.721591000 | 0.862899000 | -0.884755000 |
| B | -1.011988000 | -2.419932000 | -1.397291000 |
| B | 0.733684000 | -2.533389000 | -1.331811000 |

| | | | |
|---|---|---|---|
| B | 1.828199000 | 0.495283000 | -2.407466000 |
| B | 1.640955000 | 2.114925000 | -1.367734000 |
| B | -2.455732000 | 2.111549000 | 0.000000000 |
| B | -0.901858000 | 1.688930000 | 2.456823000 |
| B | -1.011988000 | -2.419932000 | 1.397291000 |
| B | 0.733684000 | -2.533389000 | 1.331811000 |
| B | 1.640955000 | 2.114925000 | 1.367734000 |
| B | -2.825606000 | -0.242657000 | -1.737394000 |
| B | -0.063978000 | -1.811099000 | 2.575742000 |
| B | -1.190506000 | 2.599047000 | -0.894418000 |
| B | 0.164791000 | 2.749625000 | 1.757607000 |
| B | -2.825606000 | -0.242657000 | 1.737394000 |
| B | -1.190506000 | 2.599047000 | 0.894418000 |

**TS1**

| | | | |
|---|---|---|---|
| B | 0.481682000 | 1.742921000 | -2.620731000 |
| B | -2.832012000 | 1.861233000 | 0.003102000 |
| B | -1.142922000 | 1.490505000 | -2.291117000 |
| B | 0.255302000 | -1.920038000 | 2.773964000 |
| B | 2.755739000 | -0.166718000 | 1.732642000 |
| B | 2.487777000 | 2.381236000 | 0.016005000 |
| B | -1.831841000 | -2.315686000 | 0.870559000 |
| B | 1.057011000 | -2.399985000 | -1.392730000 |
| B | 0.267393000 | -1.793687000 | -2.749304000 |
| B | -2.938024000 | -1.515800000 | 0.008570000 |
| B | -1.280614000 | -1.315230000 | -2.352951000 |
| B | -0.221898000 | 2.857268000 | 1.668544000 |
| B | -1.011556000 | 1.516437000 | 2.228024000 |
| B | -1.235057000 | -1.333031000 | 2.349709000 |
| B | 1.584776000 | -1.099232000 | 2.436298000 |
| B | 1.681470000 | 0.733523000 | -2.386287000 |
| B | -1.818608000 | -2.276101000 | -0.884436000 |
| B | 1.713915000 | 0.543667000 | 2.693067000 |
| B | -1.815218000 | 0.147651000 | 2.583930000 |
| B | -0.246006000 | 2.756789000 | -1.659142000 |
| B | -1.632513000 | 2.500242000 | -0.876509000 |
| B | -1.577592000 | 2.466788000 | 0.862108000 |
| B | 1.077110000 | -2.473209000 | 1.421747000 |
| B | 1.268498000 | 2.258533000 | -1.110720000 |
| B | 1.202580000 | 2.643957000 | 0.911999000 |
| B | -2.514389000 | -0.719653000 | 1.393325000 |
| B | -2.438973000 | 1.022641000 | 1.346063000 |
| B | 0.698180000 | 1.723948000 | 2.368648000 |
| B | 2.264757000 | 1.349313000 | 1.281144000 |
| B | 1.822519000 | -2.714141000 | 0.003130000 |

| | | | |
|---|---|---|---|
| B | 1.623769000 | -1.006222000 | -2.387986000 |
| B | -2.589992000 | -0.741804000 | -1.389584000 |
| B | -2.527254000 | 1.042447000 | -1.368883000 |
| B | 2.699797000 | 1.289506000 | -1.097570000 |
| B | -0.572095000 | -2.774868000 | 1.735430000 |
| B | -1.947029000 | 0.133538000 | -2.603546000 |
| B | 2.459265000 | -1.513458000 | 0.884142000 |
| B | 2.870382000 | -0.171212000 | -1.760637000 |
| B | -0.546165000 | -2.709861000 | -1.743899000 |
| B | 2.447837000 | -1.502208000 | -0.896116000 |
